# Oligodendrocyte-secreted ERBB3 Mediates the Competitive Uptake of Copper Ions by Tumor Cells to Promote Brain Metastasis in Lung Cancer

**DOI:** 10.7150/ijbs.127108

**Published:** 2026-03-25

**Authors:** Yuechao Yang, Yang Gao, Huanhuan Cui, Sen Li, Zhe Qi, Zhisu Wang, Deheng Li, Lei Chen, Mingtao Feng, Xiaojun Wu, Xin Chen, Bin Hao, Changshuai Zhou, Liangdong Li, Yiqun Cao

**Affiliations:** 1 Department of Neurosurgery, Fudan University Shanghai Cancer Center, Shanghai 200032, China.; 2 Department of Oncology, Shanghai Medical College, Fudan University, Shanghai 200032, China.

**Keywords:** tumor microenvironment, oligodendrocyte, lung cancer brain metastasis, ERBB3, copper transport

## Abstract

Recent evidence establishes the brain metastatic microenvironment as a key regulator of metastatic outgrowth, with oligodendrocytes being established as essential components of the brain metastatic microenvironment. Nonetheless, the mechanisms underlying oligodendrocyte-mediated brain metastasis in lung cancer await clarification. Using orthogonal experimental models spanning clinical specimens and animal models, we investigated the presence and functional roles of oligodendrocytes in lung cancer brain metastasis (LCBM). Combinatorial approaches including scRNA-seq, functional genomics, and mechanistic studies revealed ERBB3 as the critical paracrine factor mediating oligodendrocyte-tumor crosstalk. Through comprehensive analysis, we demonstrated the infiltration of oligodendrocytes in the metastatic niche of LCBM. Functional assays demonstrated that oligodendrocytes significantly enhanced the proliferation, migration, and invasion of brain metastatic cells of lung cancer. Mechanistically, oligodendrocyte-secreted ERBB3 acts as a copper chaperone that competitively mobilizes extracellular copper ions through high-affinity binding to SLC31A1 on tumor cells, thereby promoting intracellular copper accumulation. Additionally, elevated ERBB3 expression in LCBM clinical specimens correlated with significantly reduced overall survival and targeted ERBB3 suppressed lung cancer brain metastasis. Our findings establish oligodendrocyte-derived ERBB3 as a critical mediator of intercellular copper transfer via SLC31A1 binding, which coordinately drives brain metastasis progression. Therapeutic targeting of this copper signaling axis represents a promising strategy against LCBM.

## Introduction

Brain metastases are the most prevalent form of malignant brain tumors, occurring more frequently than primary malignant brain tumors such as glioblastoma (GBM) 1, 2. Distant metastasis is a leading cause of death in patients with lung cancer, with approximately 30-50% of lung cancer patients experiencing brain metastases during disease progression. Elucidating the mechanisms of metastatic colonization is critical for identifying novel therapeutic targets and improving the quality of life for lung cancer brain metastasis (LCBM) patients 3.

The interaction between cancer cells and the tumor microenvironment is crucial for metastatic colonization 4-7. Studies have indicated that macrophages can compete with tumor cells for iron ion uptake, thereby promoting meningeal metastasis 8. In the brain microenvironment, interactions between astrocytes and tumor cells have been shown to enhance tumorigenicity in LCBM 9-11. Reactive astrocytes and microglia cooperatively establish distinct niche architectures for triple-negative and HER2-positive breast cancer brain metastases 12. Furthermore, strategies targeting microglial polarization have been shown to effectively reduce LCBM 13. Pericytes actively remodel the blood brain barrier (BBB) microenvironment to promote tumor cell transmigration and establish a protective niche supporting metastatic colonization through direct cell-cell interactions 7, 14. Endothelial-expressed CD276 (B7-H3) mediates immune-tumor crosstalk, fostering an immunosuppressive microenvironment that supports metastatic progression 15.

Neurons and neural electrical activity provide trophic support for metastatic tumor cell survival 6, and metastatic cells adopt a neural-like meta-program, characterized by upregulated neuronal gene signatures 16, 17. Oligodendrocytes are widely distributed throughout the brain and exhibit functional coupling with neuronal activity 18 In various brain regions, such as the neocortical gray matter, oligodendrocytes predominate among glial cells, constituting 45%-75% of the total population, while astrocytes represent 19%-40%, and microglia are the least abundant glial cell type 19. Critically, activity-dependent myelination dynamics demonstrate their dual role in both structural support and activity-regulated circuit plasticity 18. However, at the gray-white matter junction, a predilection site for brain metastases, oligodendrocytes likely represent the predominant glial cell population 20. Although oligodendrocytes are well-characterized in central nervous system (CNS) homeostasis, their involvement in the brain metastatic niche has been largely overlooked. Multi-platform profiling of LCBM demonstrated robust oligodendrocyte present, validated in three independent clinical cohorts, suggesting their potential role in metastatic progression 21-24. In our previous study, single-cell RNA sequencing (scRNA-seq) data further showed that PSMA (FOLH1) was exclusively expressed in tumor-infiltrating oligodendrocytes. Mechanistically, oligodendrocytes upregulate PSMA expression through the ER stress pathway 25.

ERBB3 (HER3), a pseudo-kinase member of the EGFR receptor tyrosine kinase family, has emerged as a key oncogenic driver involved in tumor progression and drug resistance, across multiple malignancies 26. Its overexpression in lung and breast cancers correlated with aggressive phenotypes, including therapy resistance and poor survival 27, 28. This clinical impact was underscored by ongoing phase I/III trials of ERBB3-targeting antibody-drug conjugates (ADCs) in refractory EGFR-mutated NSCLC (U31402-A-U102/NCT03255070/NCT05338970) 29-31. Beyond cell-autonomous signaling, ERBB3 modulates the tumor immune microenvironment by promoting PD-L1-mediated immune evasion 32. It is particularly noteworthy that, in LCBM, ERBB3 was markedly upregulated (70% prevalence) compared to primary tumors, with expression levels predictive of adverse outcomes 27, 33. Despite these advances, the single-cell spatial distribution of ERBB3 within the LCBM niche and its non-canonical signaling mechanisms remain uncharacterized—a critical gap given the receptor's pleiotropic roles in metastasis and immune suppression.

In this study, we demonstrate the presence of oligodendrocytes in LCBM and identify them as major ERBB3 reservoirs. Through systematic investigation of tumor-oligodendrocyte interactions, we aimed to: (i) elucidate ERBB3-mediated survival mechanisms underlying metastatic colonization, and (ii) discover novel therapeutic targets for LCBM prevention and treatment.

## Materials and Methods

### Clinical specimens

In this study, cerebrospinal fluid (CSF) samples were systematically collected from 43 patients treated at Fudan University Shanghai Cancer Center (FUSCC) between June 2022 and October 2022, without regard to age, gender, or disease stage. The cohort comprised 17 patients with LCBM, 14 with leptomeningeal metastases (LM), 6 with gliomas, and 6 with benign conditions. The levels of secretory ERBB3 in the CSF were quantified using enzyme-linked immunosorbent assay (ELISA). Additionally, this study utilized a retrospectively collected cohort of 80 patients with LCBM. All patients underwent surgical resection of their brain metastases at FUSCC between 2016 and 2022.

Inclusion Criteria: 1) Pathologically confirmed diagnosis of primary lung adenocarcinoma; 2) Availability of formalin-fixed, paraffin-embedded tissue blocks from the surgically resected brain metastasis with sufficient material for analysis; 3) Availability of basic clinicopathological data and follow-up survival information.

Exclusion Criteria: 1) History of other primary malignancies; 2) Brain metastasis from a non-lung origin; 3) Incomplete clinical or follow-up data.

Histopathological review confirmed all primary tumors as lung adenocarcinoma. Data on key driver mutations (EGFR, ALK) were obtained from routine clinical testing records. Detailed clinicopathological and molecular characteristics of the cohort are summarized in **Table 1**.

ERBB3 expression was assessed using immunohistochemistry. Three pathologists, certified by the committee, independently assessed all immunohistochemistry (IHC) slides using a dual-phase quality control approach. ERBB3 expression was scored in a blinded manner according to the Remmele Immunoreactive Score (IRS) system, which evaluates staining intensity (ranging from 0 to 3) and the percentage of positive cells (ranging from 0 to 4). The final classification was as follows: negative (0), weak (1-4), moderate (5-8), and strong (9-12). The median score was used to divide the cases into two groups: high and low ERBB3 expression. Written informed consent was obtained from all participants prior to sample collection, and the study protocols were approved by the Ethics Committee of Fudan University Shanghai Cancer Center.

### Animal studies

All animal experiments adhered to the National Institutes of Health Guidelines for the Care and Use of Laboratory Animals and were approved by the Animal Ethics Committee of FUSCC. Male BALB/c nude mice, aged 6-8 weeks, were procured from the Institute of Experimental Animal Science and housed in a specific pathogen-free environment. To induce LCBM, mice were inoculated into the left ventricle with 100 µL of phosphate-buffered saline (PBS) containing 5 × 10^5^ luciferase-labeled H2030 and PC9 cells, utilizing a 26G needle. Prior to the procedure, mice were anesthetized with a 1.5% solution of pentobarbital sodium at a dose of 50 mg/kg. The establishment of LCBM was confirmed using *in vivo* bioluminescence imaging. For this, mice were anesthetized via intraperitoneal injection of 150 µL of a solution containing 1.5% D-fluorescein and 1.5% pentobarbital sodium. Imaging was conducted using the Xenogen IVIS system (Spectral Instrument Imaging, Lagoon X), with live image acquisition and analysis software. The study concluded four weeks post-injection. To evaluate the effect of secretory ERBB3 on LCBM development, the mice were randomly assigned to two groups. One group received intraperitoneal injections of secretory ERBB3 (1 mg/kg in 100 µL PBS) administered to H2030-BM cells one day prior to intracardiac injection, followed by weekly intraperitoneal injections of secretory ERBB3 for four weeks. The control group received only 100 µL of PBS. The occurrence of LCBM was quantitatively assessed using* in vivo* imaging, based on the detection of intracerebral bioluminescence signals. In the experimental procedure, to evaluate the impact of targeting ERBB3 on LCBM in mice, the animals were randomly divided into two groups. One group received intraperitoneal injection of Elgemtumab (HY-P99936, MedChemExpress, Monmouth Junction, NJ, USA, 1 mg/kg in 100 µL PBS), a monoclonal antibody specifically targeting ERBB3, one day prior to intracardiac injection of H2030-BM cells. Subsequently, the group was administered Elgemtumab intraperitoneally once a week for a total of four weeks. The control group was injected with 100 µL PBS without any treatment. Hematoxylin and eosin (H&E) staining was used to evaluate the differences in tumor formation rate and tumor burden between the two groups.

### Isolation and purification of brain-metastatic cells

Mice exhibiting LCBM were identified and selected through bioluminescence imaging. Upon euthanasia, brain metastatic lesions were meticulously excised under sterile conditions. The collected tissues were then dissected and submerged in RPMI-1640 or DMEM containing 0.2% collagenase I. This mixture was incubated at 37 °C in a humidified environment for approximately 1 h, with gentle agitation every 20 min. Following incubation, the cells were subjected to low-speed centrifugation (1,200 rpm for 5 min), resuspended in 2 mL of 0.25% trypsin, and further incubated for an additional 10 min. The cells were then centrifuged again and resuspended in RPMI-1640 supplemented with 10% FBS and 1% penicillin-streptomycin. The resuspended cells were cultured and allowed to adhere in 25-cm² culture flasks. Brain-metastatic cells were selected using puromycin, and the isolated metastatic cell lines (H2030-BM1, PC9-BM1) were expanded and cultured. These cells were subsequently re-inoculated into mice, resulting in the establishment of the H2030-BM and PC9-BM cell lines.

### Cell culture and reagents

The human NSCLC cell lines H2030 (RRID: CVCL_1512), PC9 (RRID: CVCL_B260), and H1299 (RRID: CVCL_0060), as well as the human oligodendrocyte cell line MO3.13 (RRID: CVCL_D357), were obtained from the American Type Culture Collection (ATCC, Manassas, VA, USA). These cells were cultured in either Dulbecco's Modified Eagle Medium (DMEM) or RPMI-1640 medium, supplemented with 10% fetal bovine serum (FBS) and 1% penicillin/streptomycin (Gibco, Invitrogen, USA). All cell cultures were maintained at 37°C in a 5% CO2 atmosphere. For bioluminescent tracking, H2030 and PC9 cells underwent stable transfection with firefly luciferase, accompanied by an antibiotic resistance gene (Genechem, Shanghai, China). Puromycin, sourced from GeneChem, was used for selection, with all reagents stored and handled in accordance with the manufacturer's guidelines.

### Cell transfection

To downregulate the expression of SLC31A1, two small interfering RNAs (siRNAs) targeting SLC31A1, siSLC31A1-1: 5'-AUUCUUAAAGCCAAAGUAGAA-3' and siSLC31A1-2: 5'-AAAACACUGCCACAAAAGCUC-3' (presented as the sense strand), along with a negative control (NC), were synthesized by Tsing Ke (Shanghai, China). Transfection was performed according to the manufacturer's protocol for Lipofectamine 3000 (Invitrogen, Carlsbad, California, USA).

### Cell viability CCK-8 assay

Cell viability alterations were assessed through the Cell Counting Kit-8 (CCK-8) assay. Specifically, Brain metastatic cells, post 24-hour gene transfection, were plated in 96-well plates at a concentration of 3000 cells per well, utilizing 100 µl of complete medium, and incubated at 37 °C. Upon the conclusion of each experimental period, 10 µl of CCK-8 reagent (Yeason, Shanghai, China) was introduced to each well, followed by an additional 2-hour incubation at 37 °C. Subsequently, the optical density (OD450) was quantified employing microcoder. Each assay was conducted in triplicate and the procedure was replicated a minimum of three times.

### Transwell invasion assay

To conduct the invasion assay, a 50 µL aliquot of Matrigel, pre-diluted in ice-cold serum-free medium at an 8:1 ratio, was applied to coat the filter. The transwell chambers were then incubated at 37 °C for 1 hour. Afterward, the upper chamber received a seeding of 4 × 10^4^ cells in 100 µL of FBS-free McCoy's 5A medium, while the lower chamber was supplied with 500 µL of 10% FBS medium. The assay continued for 48 hours at 37 °C. Cells remaining on the upper filter surface were then removed using a cotton swab, while those that had migrated to the reverse side were fixed with 4% paraformaldehyde at room temperature for 20 minutes, followed by staining with 0.1% crystal violet for an additional 20 minutes. Excess crystal violet was washed off with PBS, and the migrated cells were examined under a microscope at 200x magnification across five randomly selected fields and subsequently counted.

### Wound-healing migration assay

Cells were seeded in 6-well plates, each containing 2 ml of culture medium supplemented with 10% FBS, and incubated for 24 h. Upon reaching 95-100% confluency, the cells were treated with 2 µg/mL mitomycin C (Sigma-Aldrich, #M4287) for 2 hours at 37 °C to inhibit proliferation. A sterile 200 µl pipette tip was employed to induce vertical scratches through the cell monolayers. The detached cells were then rinsed away using PBS, and the wells were replenished with 2 ml of serum-free medium for a subsequent 48-hour incubation period. Cell migration was monitored at the onset and conclusion of this period via an Olympus light microscope, capturing images from three arbitrary fields per experimental variant at a 40x magnification.

### Tissue immunohistochemistry and immunofluorescence staining

Hematoxylin and eosin (H&E), immunohistochemistry, and immunofluorescence staining were conducted following standard proceduress using ERBB3 antibodies (Cat. #12708S; Cell Signaling Technology; 1:250), OLIG2 antibodies (Cat. #13999-1-AP; Proteintech; 1:500), MBP antibodies (Cat. #ET1702-15; HUABIO; 1:500) and SLC31A1 antibodies (Cat. #67221-1-Ig; Proteintech; 1:100). The expression levels of ERBB3 were scored using the IHC scoring method by a pathologist and an investigator.

### Western blotting and co-immunoprecipitation (co-IP)

For western blotting analysis, cells were lysed using RIPA buffer supplemented with a protease and phosphatase inhibitor cocktail, and the protein concentration was quantified using the bicinchoninic acid (BCA) protein assay. Equal amounts of protein were subjected to SDS-PAGE and then transferred onto polyvinylidene fluoride (PVDF) membranes. The membranes were blocked with 5% bovine serum albumin (BSA) in Tris buffered saline Tween (TBST), followed by incubation with primary antibodies: ERBB3 (Cat. #12708S; Cell Signaling Technology; 1:1,000), p-PI3K (Cat. #4292S; Cell Signaling Technology; 1:1,000), p-Akt (Cat. #66444-1-Ig, Proteintech; 1:5,000), p-mTOR (Cat. #AF3308; Affinity; 1:1,000), JAK1 (Cat. #AF5012; Affinity; 1:1,000), STAT1 (Cat. #AF6300; Affinity; 1:1,000), STAT2 (Cat. #AF6342; Affinity; 1:1,000), STAT3 (Cat. #AF6294; Affinity; 1:1,000), SLC31A1 (Cat. #67221-1-Ig, Proteintech; 1:5,000), Vimentin (Cat. #60330-1-Ig; Proteintech; 1:4,000), E-cadherin (Cat. #60335-1-Ig; Proteintech; 1:5,000), ZEB1 (Cat. #DF7414; Affinity; 1:1,000), N-cadherin (Cat. #66219-1-Ig; Proteintech; 1:5,000), PD-L1 (Cat. #DF6526; Affinity; 1:1,000), and β-actin (Cat. #81115-1-RR; Proteintech; 1:5,000) overnight at 4 °C. This was followed by incubation with horseradish peroxidase-conjugated secondary antibodies (Cat. #SA00001-1/SA00001-2; Proteintech; 1:2,000). The protein bands were visualized using an enhanced chemiluminescence (ECL) reagent (Share Biotechnology, Shanghai, China) and the intensity was analyzed with image processing software.

For co-IP experiments, cell lysates were pre-cleared using protein A/G-agarose beads and subsequently incubated with the antibody of interest conjugated to protein A/G-agarose beads overnight at 4 °C. The beads were thoroughly washed to eliminate non-specific binding, and the bound proteins were eluted by heating in SDS sample buffer. This was followed by SDS-PAGE and Western blotting analysis to detect the co-immunoprecipitated proteins.

### ELISA

To assess the concentrations of ERBB3 in the cellular supernatant, we utilized an ELISA technique with a commercially available kit (Bio-Swamp, China), adhering to the manufacturer's protocols. The samples were directly applied to each well of a 96-well plate pre-coated with an anti-human ERBB3 antibody provided in the kit. Subsequently, absorbance readings were recorded at a wavelength of 450 nm using a microplate spectrophotometer (Molecular Devices VersaMax, Silicon Valley, USA). ERBB3 levels were subsequently determined based on a standard curve.

### The 5D-label-free quantitative proteomics

5D-label-free quantitative proteomics analysis was performed by GeneChem (Shanghai, China) to evaluate changes in the complete protein profile of the oligodendrocyte cell line supernatant. Briefly, MO3.13 and H2030-BM cells were cultured, and proteins from the supernatant of MO3.13 cells co-cultured with H2030-BM were subjected to trypsin digestion to yield polypeptides. The serum-free medium of MO3.13 served as the control. Rigorous quality control measures were implemented to ensure accuracy. Subsequently, GeneChem aided with high-performance liquid chromatography (HPLC) fractionation, liquid chromatography-mass spectrometry (LC-MS/MS) analysis, database searches, bioinformatics analysis, and protein-protein interaction mapping. The false discovery rate (FDR) for protein identification and peptide spectral match (PSM) was set at 5%. A *p*-value ≤ 0.05 and a fold-change cutoff in quantitative protein ratios exceeding 1.5 or less than 1/1.5 were considered statistically significant.

### Measurement of copper ions

The concentration of copper ions in cell lines was measured using the Cell Copper (Cu) Colorimetric Assay Kit (Elabscience, Wuhan, China) following the manufacturer's instructions. This kit was utilized to determine the copper ion levels in MO3.13, PC9, PC9-BM, H2030, and H2030-BM cells according to the provided protocol.

### Available RNA-sequencing

RNA sequencing was conducted on all four groups (three biological replicates per group): H2030-BM, H2030-BM-co-culture, MO3.13, and MO3.13-co-culture. After 48 hours of co-culture with conditioned medium, total RNA was extracted from the final three samples for RNA sequencing analysis. The raw RNA-seq reads were processed using a standardized workflow for RNA-sequencing analysis. ScRNA-seq datasets were obtained from the National Center for Biotechnology Information Gene Expression Omnibus (NCBI GEO) database (GSE131907). A subset of this dataset was selected, comprising 10 cases with a total of 33,280 cells, including six lung cancer samples and four brain metastasis samples. The scRNA-seq data were analyzed using the Seurat R package (version 4.3.0).

### Statistical analysis

Statistical analysis of the data was performed using GraphPad Prism 9.0 and SPSS 19.0 software (SPSS, Chicago, IL, USA). The data were recorded as mean ± standard error of the mean. The ANOVA test was employed to identify differences between the two groups, while the Student's t-test was utilized to determine variations between the two groups. The association between ERBB3 expression and clinicopathological traits was analyzed using the Chi-squared (χ2) test, with a *p*-value less than 0.05 considered statistically significant.

## Results

### Comprehensive evaluation of oligodendrocyte spatial distribution patterns in brain metastatic lesions

**Figure 1:**
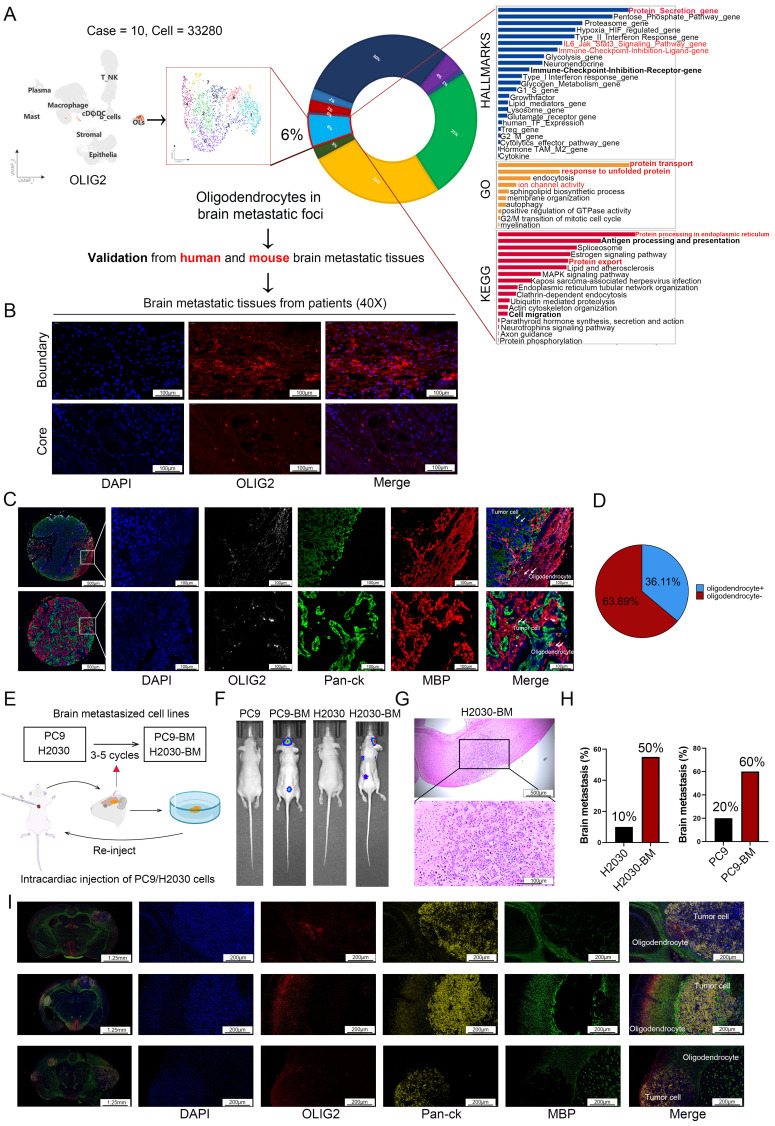
** Assessment of oligodendrocyte distribution in brain metastases.** (A) The proportion of oligodendrocyte distribution in LCBM was analyzed using single-cell transcriptome sequencing data. (B) Evaluation the distribution of oligodendrocytes in LCBM using immunofluorescence. (C-D) Multiple immunofluorescence staining was used to evaluate the distribution of oligodendrocytes in LCBM tissue microarray. (E) Schematic representation of the* in vivo* selection process. (F-G) LCBM were determined by bioluminescence imaging and H&E staining. (H) Bar chart showing the brain metastasis rate of primary cells and brain metastatic cells. (I) Evaluation the distribution of oligodendrocytes in LCBM in mice using immunofluorescence. All experiments were repeated three times (* *p* < 0.05, ** *p* < 0.01, *** *p* < 0.001, ns > 0.05).

While scRNA-seq studies have implicated oligodendrocyte in LCBM, their functional significance and underlying mechanisms remain poorly understood 21-24. To systematically assess the cellular composition and quantify oligodendrocyte populations in the LCBM tumor microenvironment, we conducted in-depth analysis of scRNA-seq data from the GSE131907 dataset. Our findings indicated that oligodendrocytes accounted for approximately 6% of the cell population within the tumor sites (**Figure 1A**). Subsequently, we investigated the characteristics of oligodendrocytes in LCBM. Hallmark enrichment analysis indicated that oligodendrocytes were notably involved in protein secretion and immune checkpoints related pathways. GO and KEGG analyses further identified significant enrichment of pathways associated with protein transport, response to unfolded proteins, and ion homeostasis (**Figure 1A**). These enrichment profiles suggest a potential role for oligodendrocytes in these cellular processes in LCBM.

Subsequently, we employed immunofluorescence staining to systematically evaluate the spatial distribution of oligodendrocytes in LCBM. Our analysis revealed a distinct expression pattern of OLIG2, a specific oligodendrocyte lineage marker, with robust immunoreactivity observed at the metastatic border zone, while demonstrating minimal expression within the tumor core region (**Figure 1B**). Next, we utilized a tissue microarray (TMA) platform encompassing 36 LCBM specimens obtained from the FUSCC cohort to validate the aforementioned findings through systematic immunohistochemical analysis (**Figure 1C**). All samples were stained with DAPI, OLIG2 (Aqua), MBP (Cy5), and Pan-ck (SpGreen) (**Figure. 1C**). Mature oligodendrocytes were identified by co-expression of OLIG2 and MBP. The presence of oligodendrocytes was observed in 13/36 LCBM, mainly at the border of the tumor (**Figure 1D**). Furthermore, we implemented multiplex immunofluorescence on two whole-tissue sections, coupled with spatial transcriptomic verification in lung cancer brain metastasis samples, to definitively confirm the oligodendrocyte expression pattern (**Figure S1A-B**).

### Establishment of cells model for lung cancer brain metastasis

Due to the marked heterogeneity between LCBM and primary lung cancer lesions, coupled with the technical limitations of establishing mouse LCBM models using *in vitro*-cultured cells, we generated lung cancer cell lines with enhanced LCBM tropism for subsequent experimental studies. Brain-tropic subpopulations were generated from NSCLC cell lines PC9 and H2030 through serial *in vivo* selection (**Figure 1E**). After several rounds of cycling screening, we developed high brain metastasis cell lines, H2030-BM, which demonstrated a 50% metastasis rate, and PC9-BM, with a 60% metastasis rate (**Figure 1H**). Both *in vivo* fluorescence imaging and histopathological analysis revealed significantly enhanced intracranial tumor formation capacity in the LCBM variants compared to their parental cell lines (**Figure 1F-G**). Immunofluorescence analysis of brain sections from these nude mouse models revealed that oligodendrocytes were present within the tumor microenvironment of established metastases and were predominantly localized at their boundaries (**Figure 1I**). This spatial distribution pattern is consistent with that observed in patient samples.

Furthermore, transcriptomic profiling of H2030 parental cells versus their brain-metastatic derivatives (H2030-BM) (**Figure S1C-D**), with particular focus on EMT (epithelial-to-mesenchymal transition)-related genes, demonstrated that both PC9-BM and H2030-BM sublines had acquired molecular signatures consistent with epithelial-mesenchymal transition (**Figure S1E-F**). Functional characterization of the metastatic variants revealed significantly enhanced invasive capacity in both PC9-BM and H2030-BM cells compared to their parental counterparts (**Figure S1G**).

### Oligodendrocytes enhanced proliferation, migration, and invasion of lung cancer brain metastatic cells* in vitro*

**Figure 2:**
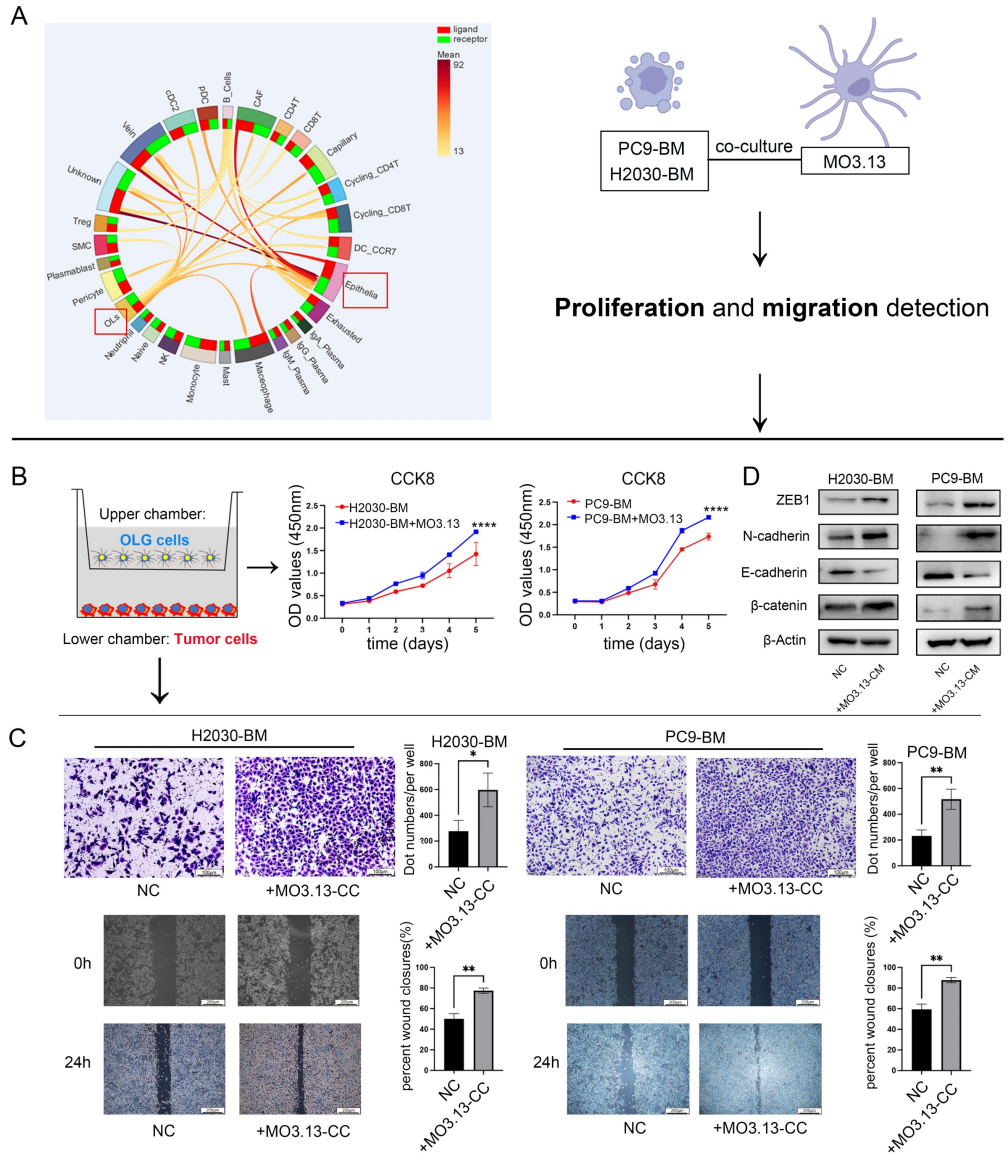
** Effect of oligodendrocytes on the function of lung cancer brain metastatic cells.** (A) Prediction of cell interactions in the microenvironment of LCBM based on single-cell transcriptome sequencing data analysis. (B-C) Oligodendrocytes and lung cancer brain metastatic cells were co-cultured in a non-contact manner, then subjected to the Transwell, wound-healing and CCK8 assays. Magnification, × 200. (D) Expression difference of EMT pathway protein in tumor cells after co-culture. All experiments were repeated three times (* *p* < 0.05, ** *p* < 0.01, *** *p* < 0.001, ns > 0.05).

scRNA-seq data analysis revealed reciprocal tumor cell-oligodendrocyte interactions via multiple ligand-receptor pairs (**Figure 2A**). To investigate oligodendrocyte-mediated modulation of tumor cell dynamics, we conducted functional analyses using CCK-8 proliferation assays, transwell invasion chambers, and scratch wound healing assays. Oligodendrocyte-conditioned media markedly potentiated the proliferation, invasion, and migration of brain-metastatic lung cancer cells (**Figure 2B-C**).

Based on the presented findings, we hypothesized that responsive oligodendrocyte aid in the progression of tumor cells through the EMT mechanism. To validate this hypothesis, we exposed lung cancer brain metastatic tumor cells to oligodendrocyte-conditioned media and evaluated the expression of EMT marker proteins using western blotting. Our analysis showed an upregulation of N-cadherin and β-catenin, and a downregulation of E-cadherin in the treated cells compared to the control group. Additionally, there was an elevated expression of the EMT-related transcriptional repressor ZEB1 (**Figure 2D**). Collectively, these molecular changes are consistent with the acquisition of a mesenchymal-like phenotype, supporting the hypothesis that oligodendrocyte-derived factors may promote a pro-metastatic state in tumor cells.

### Oligodendrocyte secreted ERBB3 mediated its interaction with lung cancer brain metastatic tumor cells

**Figure 3:**
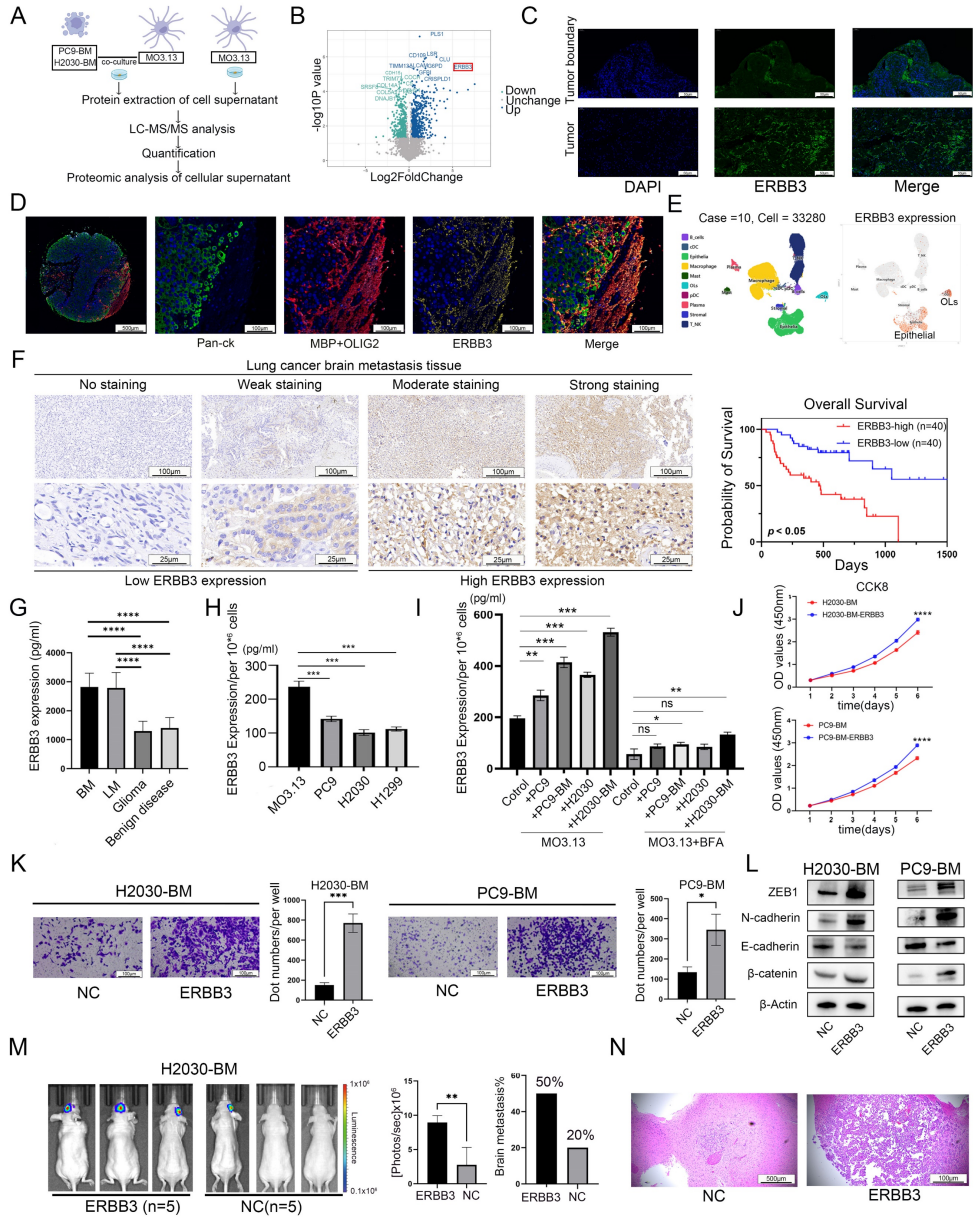
** The secreted ERBB3 protein may be involved in the interaction between oligodendrocytes and tumor cells.** (A) Schematic diagram of cellular superalbumin proteomics. (B) Volcano diagram showing differential proteins in the cell supernatant. (C) ERBB3 expression and distribution assessed using immunofluorescence in LCBM. (D) Multiple immunofluorescence staining was used to analyze the expression pattern of ERBB3 in LCBM. (E) Distribution of ERBB3 expression in scRNA-seq data. (F) Correlation between ERBB3 expression and prognosis in LCBM evaluated by immunohistochemistry. (G) Expression level of ERBB3 in CSF. (H) ERBB3 secretion and expression in various cell lines. (I) Changes in ERBB3 secretion following co-culture of oligodendrocytes and lung cancer cells. (J) The effect of secretory ERBB3 on cell proliferation was investigated using CCK-8 assay. (K) The invasion function of tumor cells was evaluated by transwell assay. (L) Differential expression of EMT pathway proteins. (M) Brain metastases were determined by bioluminescence imaging and a histogram displayed the incidence of brain metastases across different groups. (N) Brain sections were stained by H&E. All experiments were repeated three times. All experiments were repeated three times (* *p* < 0.05, ** *p* < 0.01, *** *p* < 0.001, ns > 0.05).

Considering the significant enrichment of protein-secretion hallmarks in infiltrating oligodendrocytes within brain metastases (**Figure 1A**), and the paracrine signaling from oligodendrocytes to tumor cells through multiple ligand-receptor pairs (**Figure 2A**), as evidenced by the scRNA-seq data analysis, we performed a quantitative proteomic analysis of the supernatants from oligodendrocytes co-cultured with lung cancer brain metastatic cells. The serum-free supernatant from oligodendrocytes cultured for 48 h served as the control group (**Figure 3A**). The resulting volcano plot showcased the differential proteins, with a notable increase in ERBB3 secretion following co-culture (**Figure 3B**). Subsequent clustering and differential gene analysis identified significant alterations in 986 proteins in the supernatant: 649 were upregulated, and 337 were downregulated (**Figure S2A**). These differential proteins were predominantly localized in the cytoplasm, with additional proteins distributed in the extracellular space and plasma membrane (**Figure S2B**). GO and KEGG enrichment analyses of the differential proteins revealed their involvement in metabolic and cancer-related pathways (**Figure S2C-D**). Furthermore, Gene Set Enrichment Analysis (GSEA) indicated that these differential proteins play a critical role in cancer development and the EMT pathways (**Figure S2E**).

Tomasich *et al.* and Kodack *et al.* reported that compared with primary tumors, ERBB3 expression was significantly upregulated in LCBM 27, 34. Subsequently, immunofluorescence staining results revealed that spatially, ERBB3 was mainly expressed at the LCBM tumor border, and at the cellular level, it was notably highly expressed in oligodendrocytes (**Figure 3C-D**). While, scRNA-seq data revealed that, in transcriptome level, ERBB3 was primarily expressed both in oligodendrocytes and tumor cells in LCBM (**Figure 3E**).

We next performed immunohistochemical staining for ERBB3 in a cohort of 80 LCBM patients. Based on staining intensity, samples were categorized into high- and low-expression groups to evaluate the association between ERBB3 levels and clinicopathological features. Our analysis revealed that high ERBB3 expression was correlated with poorer prognosis in LCBM patients (**Figure 3F, Table 1**). Further stratification by EGFR mutation status showed that among EGFR-mutant cases, high ERBB3 expression was associated with significantly worse survival. In contrast, among EGFR wild-type patients, ERBB3 expression did not show a statistically significant prognostic association (**Figure S3A**). Finally, both univariate and multivariate Cox regression analyses confirmed that elevated ERBB3 expression serves as an independent prognostic factor for poor outcome in LCBM patients (**Table 2**).

Given the significant increase in ERBB3 secretion by oligodendrocytes after *in vitro* co-culture with tumor cells (**Figure 3B**), and considering that CSF is a key component of the brain microenvironment that reflects secreted cellular proteins, we collected CSF samples from patients with various brain tumors at FUSCC. Our findings indicated that, in comparison to patients with gliomas or benign brain conditions, those with LCBM or lung cancer leptomeningeal metastases (LCLM) exhibited significantly elevated levels of secretory ERBB3 (**Figure 3G**). This differential elevation suggests that secretory ERBB3 could be a distinctive feature of LCBM. Furthermore, we identified disparities in ERBB3 expression between oligodendrocytes and lung cancer cell lines. Specifically, when comparing an equal number of cells, oligodendrocytes secreted a higher level of ERBB3 protein than lung cancer cells did (**Figure 3H**). Finally, following a 48h incubation of oligodendrocytes with the conditioned medium from lung cancer and brain metastatic cell lines, the secretion level of ERBB3 significantly rose (**Figure 3I**). Nevertheless, the addition of the protein transport inhibitor brefeldin A (BFA) significantly suppressed this secretion (**Figure 3I**).

### Secreted ERBB3 protein facilitated LCBM both* in vitro* and *in vivo*

To mimic the impact of oligodendrocyte-secreted ERBB3 on LCBM, we treated tumor cells (H2030-BM and PC9-BM) with recombinant secretory ERBB3 protein and carried out a series of experiments to assess alterations in cellular dynamics. Like the effects observed in co-culture with oligodendrocytes, secretory ERBB3 promoted the proliferation, migration, and invasion of lung cancer brain metastatic cells (**Figure 3J-K**). Moreover, at the molecular level, secreted ERBB3 upregulated the expression of key markers associated with an EMT-like phenotype (**Figure 3L**).

In* in vivo* experiments, lung cancer brain metastatic tumor cells were injected into the ventricles of mice. The incidence of brain metastases and tumor volume were significantly higher in the group treated with the recombinant ERBB3 protein compared to the control group (**Figure 3M-N**).

### Oligodendrocyte-derived ERBB3 promoted tumor progression via activating PI3K-Akt-mTOR pathway in tumor cells and stimulating JAK-STAT-PDL1 signaling cascade in oligodendrocytes

**Figure 4:**
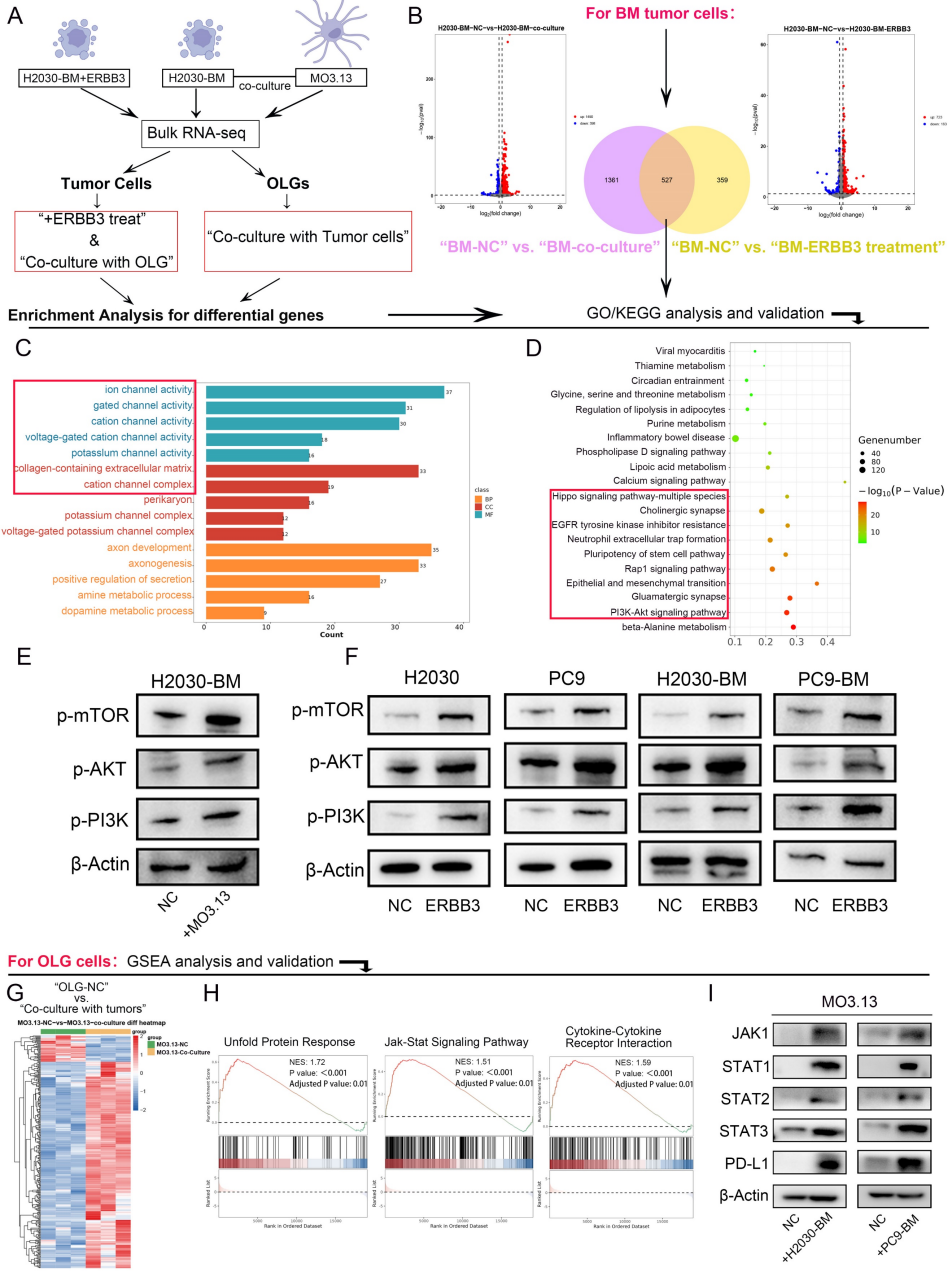
** PI3K-Akt-mTOR signaling pathway in tumor cells and JAK-STATs-PDL1 signaling axis in oligodendrocytes were activated respectively after co-culture or secretory ERBB3 treated.** (A) Flowchart depicting the cell sequencing process. (B) Volcano diagram showing differential genes in tumor cells. (C-D) GO and KEGG enrichment analysis of differential genes in tumor cells. (E) Validation of key proteins in the signaling pathway in tumor cells stimulated by the conditioned medium (CM)of oligodendrocytes. (F) Validation of key proteins in the signaling pathway in tumor cells stimulated by ERBB3 recombinant protein. (G) Cluster analysis of differential genes in oligodendrocytes. (H) GSEA enrichment analysis of differential genes in oligodendrocytes co-cultured with tumor cells. (I) Validation of key proteins in the signaling pathway in oligodendrocytes after co-culture with tumor cells. All experiments were repeated three times.

Transcriptomic sequencing was further performed on both co-cultured tumor cells and oligodendrocytes, as well as on tumor cells treated with the secretory ERBB3 recombinant protein. Differential gene analysis and pathway enrichment analysis were conducted on these datasets, followed by subsequent analyses of the intersecting differential genes (**Figure 4A-B**). GO analysis indicated that secretory ERBB3 predominantly affected biological processes related to ion channel activity (**Figure 4C**), while KEGG enrichment analysis revealed that it stimulated pathways involved in EGFR tyrosine kinase inhibitor resistance, PI3K-Akt-mTOR signaling, and EMT in tumor cells (**Figure 4D**). The PI3K-Akt-mTOR pathway was also observed to be activated in tumor cells that were co-cultured with oligodendrocytes (**Figure 4E**). Further assessment of PI3K-Akt-mTOR pathway proteins showed significant activation of this pathway when tumor cells were treated with the ERBB3 recombinant protein (**Figure 4F**).

Transcriptome sequencing data were also analyzed in oligodendrocytes that were co-cultured with tumor cells (**Figure 4G**). GSEA analysis suggested potential activation of the UPR (unfolded protein response), JAK-STAT, and Cytokine-Cytokine Receptor Interaction pathways following co-culturing (**Figure 4H**). Confirmatory tests in oligodendrocytes exposed to conditioned medium showed activation of the JAK-STAT pathway and increased PD-L1 expression after co-culturing with lung cancer brain metastatic cells (**Figure 4I**). Leveraging our sc RNA-seq data from LCBM samples (OMIX007088), we grouped the samples based on the expression levels of ERBB3 in oligodendrocytes. Comparative immune profiling revealed that samples harboring ERBB3-high oligodendrocytes contained a reduced proportion of cytotoxic T cell subsets (including CD8_GZMK and CD8_GZMH cells) and an increased proportion of regulatory T cells (Tregs), relative to their ERBB3-low counterparts. To validate this association at the protein level and in an independent cohort, we performed multiplex immunofluorescence on a LCBM tissue. Consistently, lesions with high ERBB3 expression exhibited significantly elevated PD-L1 expression specifically, concomitant with a reduced density of infiltrating CD8⁺T cells. In contrast, ERBB3-low lesions displayed lower PD-L1 expression and a higher CD8⁺T cell density (**Figure S4A-B**).

### Oligodendrocyte-derived ERBB3 mediated copper ion transfer from oligodendrocytes to tumor cells via SLC31A1 binding on tumor cell surfaces

**Figure 5:**
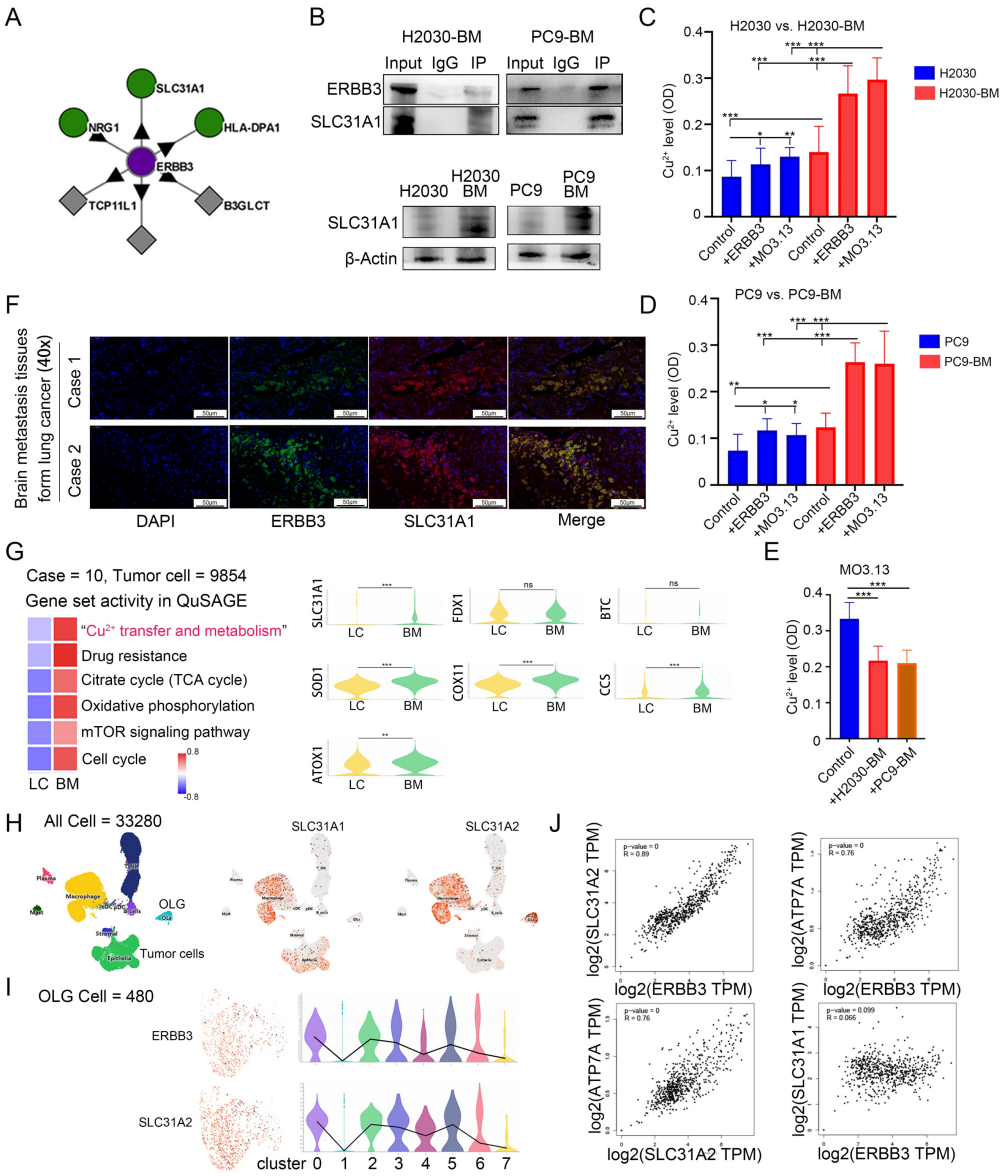
** ERBB3 specifically binds SLC31A1 to promote the transfer of copper ions into tumor cells.** (A) Bioinformatics were employed to explore the interaction between ERBB3 and SLC31A1 using public databases. (B) co-IP was used to detect the interaction between ERBB3 and SLC31A1, and western blotting was used to detect the expression of SLC31A1 in brain metastasis cell lines. (C-E) The concentration of copper ions in tumor cells and oligodendrocytes was quantified. (F) Immunofluorescence verified the interaction between ERBB3 and SLC31A1. (G) The expression difference of genes related to the enrichment pathway in metastatic tumor and primary tumor was analyzed by use of scRNA-seq data. (H-I) The cellular distribution of ERBB3, SLC31A1, and SLC31A2 expression was examined using single-cell transcriptome sequencing data. (J) Bioinformatics to explore the expression correlation between ERBB3 and copper transport-related proteins in normal brain tissue from public databases. All experiments were repeated three times (* *p* < 0.05, ** *p* < 0.01, *** *p* < 0.001, ns > 0.05).

To further elucidate the mechanism by which secretory ERBB3 activates the PI3K-Akt signaling pathway in tumor cells, we used the BioPlex Interactome database 35-37 to predict potential interacting proteins of ERBB3 (**Figure 5A**). Considering that secretory ERBB3 predominantly influences biological processes related to ion-channel activity in tumor cells (**Figure 4C**), we identified the copper-ion transporter SLC31A1 as a candidate for further validation. Subsequent co-IP assays confirmed the specific binding affinity between ERBB3 and SLC31A1 (**Figure 5B**). Notably, SLC31A1 expression was higher in brain metastasis cell lines than in primary lung cancer cell lines (**Figure 5B**). Guo *et al.* have demonstrated that elevated copper ion levels in tumors can promote tumor progression by activating the PDK1-Akt oncogenic pathway 38. Following this, we measured the copper ion concentration in lung cancer brain metastatic cells after co-culture with oligodendrocytes and exposure to recombinant ERBB3 protein. Our results showed a significant increase in copper ion levels within tumor cells following co-culture with oligodendrocytes and treatment with recombinant ERBB3 protein (**Figure 5C-D**). Concurrently, we assessed the copper ion concentration in oligodendrocytes after co-culture with lung cancer brain metastatic cells, revealing a marked decrease in copper ion levels in oligodendrocytes (**Figure 5E**). Furthermore, immunofluorescence analysis confirmed the spatial co-localization of ERBB3 and SLC31A1 in lung cancer brain metastatic tissues, suggesting a potential interaction between these two proteins (**Figure 5F**).

Upon analyzing scRNA-seq data from LCBM, we discovered that, in comparison to primary lung cancer tumor cells, brain metastatic tumor cells exhibit significant enrichment in gene sets linked to copper ion metabolism, drug resistance, the tricarboxylic acid cycle (TCA) cycle, oxidative phosphorylation, mTOR signaling, and the cell cycle (**Figure 5G**). Nevertheless, the expression of FDX1, a crucial gene regulating 'copper-induced cell death' 39, remains unchanged in brain metastatic cells (**Figure 5G**).

Furthermore, we investigated the correlation between the expression of ERBB3 and the copper ion transporters SLC31A1 and SLC31A2 at the single-cell level. Our findings uncovered a parallel expression pattern between ERBB3 and SLC31A2 specifically in oligodendrocytes (**Figure 5H-I**). Additionally, in healthy brain tissues 40, 41, ERBB3 expression was closely tied to intracellular copper transport and efflux molecules, including SLC31A2 and ATP7A (**Figure 5J**). It's worth noting that the expression of SLC31A2 and ATP7A is cell-type specific, predominantly observed in glial cells within brain tissues 42, 43. However, a clear correlation between ERBB3 expression and copper uptake molecules, such as SLC31A1, was not observed (**Figure 5J**).

### Targeting ERBB3 inhibited LCBM* in vitro* and* in vivo*

**Figure 6:**
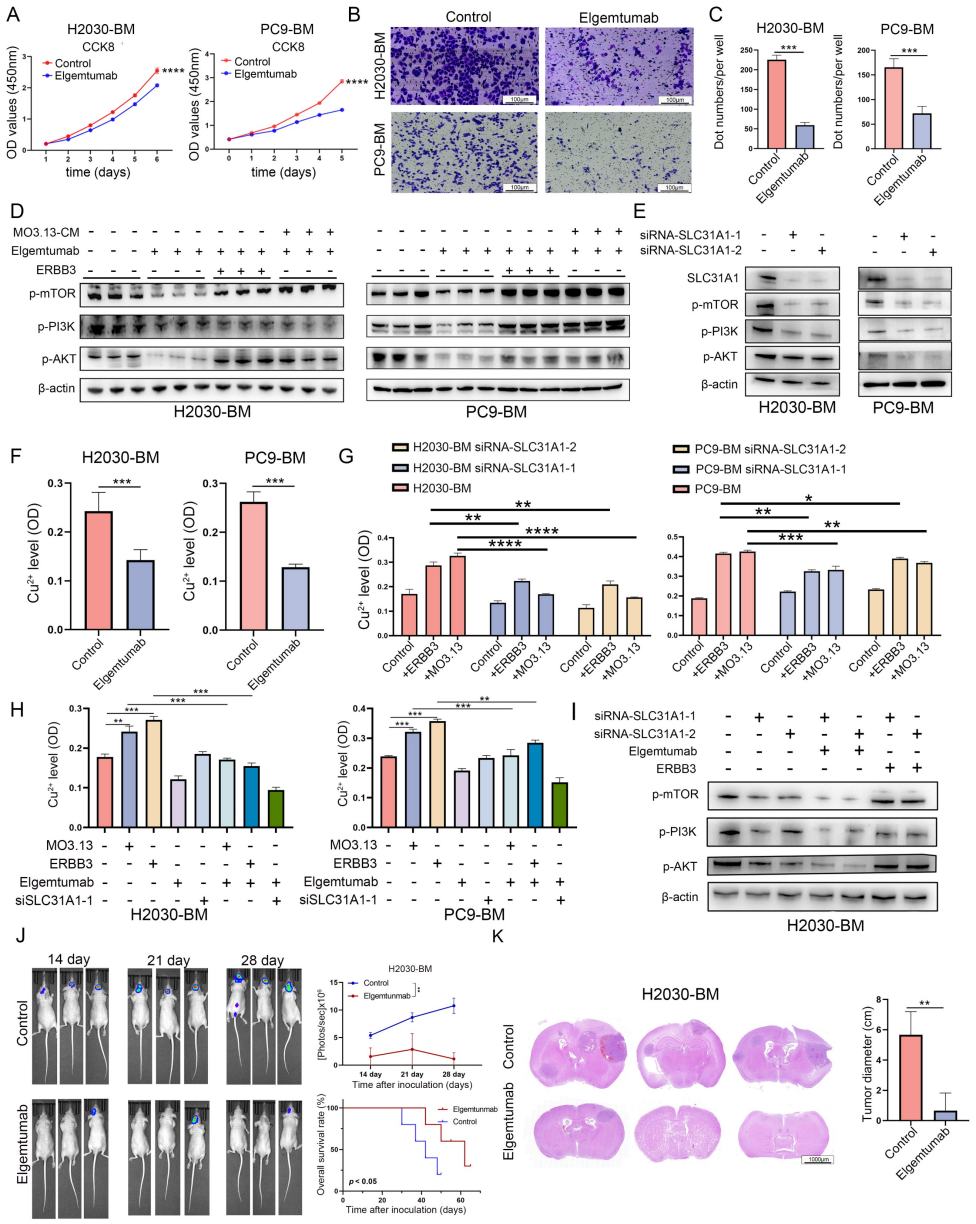
** Targeting ERBB3 to suppress LCBM* in vitro* and* in vivo*.** (A) The effect of Elgemtumab on cell proliferation was investigated using CCK-8 assay. (B-C) The invasion function of H2030-BM and PC9-BM was evaluated by transwell assay. (D-E) The expression of PI3K-Akt-mTOR pathway proteins was detected using western blotting. (F-H) Intracellular copper ion enrichment was detected in H2030-BM and PC9-BM. (I) The expression of PI3K-Akt-mTOR pathway proteins was detected using western blotting. (J) Brain metastases were determined by bioluminescence imaging. (K) Brain sections were stained by H&E. All experiments were repeated three times (* *p* < 0.05, ** *p* < 0.01, *** *p* < 0.001, ns > 0.05).

We further substantiated the therapeutic efficacy of targeting ERBB3 in treating LCBM through a comprehensive series of in *vitro* and in* vivo* experiments. Initially, H2030-BM and PC9-BM cells were treated with Elgemtumab, an anti-ERBB3 monoclonal antibody, to assess alterations in cellular proliferation and invasiveness. The results demonstrated a notable suppression of both proliferation and invasion in H2030-BM and PC9-BM cells following Elgemtumab treatment (**Figure 6A-C**). Notably, Elgemtumab significantly inhibited the PI3K-Akt-mTOR signaling pathway in H2030-BM and PC9-BM cells and reversed the pathway activation induced by ERBB3 recombinant protein stimulation and co-culture with oligodendrocytes (**Figure 6D**). Subsequently, siRNA-mediated knockdown of SLC31A1 was implemented in H2030-BM and PC9-BM cells, with knockdown efficiency confirmed at the protein level. This knockdown also curtailed activation of the PI3K-Akt-mTOR pathway (**Figure 6E**).

Measurement of intracellular copper levels revealed a decrease in copper ion concentration in cells treated with Elgemtumab (**Figure 6F**). Assessment of copper content in SLC31A1-knockout cell lines demonstrated that knockout of SLC31A1 significantly reversed copper accumulation induced by ERBB3 or oligodendrocyte stimulation (**Figure 6G**). Subgroup analysis indicated that both MO3.13 and ERBB3 elevated intracellular copper levels in tumor cells. Elgemtumab treatment reversed the copper accumulation caused by MO3.13 and ERBB3, while combined knockout of SLC31A1 and Elgemtumab treatment led to a more pronounced reduction in intracellular copper ions (**Figure 6H**). Furthermore, combining SLC31A1 knockdown with Elgemtumab treatment yielded a more pronounced inhibition of the PI3K-Akt-mTOR pathway, and SLC31A1 knockdown effectively reversed the signaling activation caused by ERBB3 recombinant protein stimulation (**Figure 6I**).

In animal experiments, tumor growth was tracked over time by in vivo fluorescence imaging at days 14, 21, and 28 post-injection. The resulting growth curves showed that the fluorescence intensity in the control group was significantly higher than that in the Elgemtumab-treated group (**Figure 6J**). Consistently, Kaplan-Meier survival analysis demonstrated that mice in the control group had a markedly shorter median survival following intracerebral tumor cell injection than those in the Elgemtumab-treated group (**Figure 6J**). Furthermore, we directly compared the brain metastatic burden between the control and Elgemtumab-treated cohorts. Quantitative assessment confirmed that the tumor load in the brains of Elgemtumab-treated mice was substantially lower than that in the control group (**Figure 6K**).

**Figure 7:**
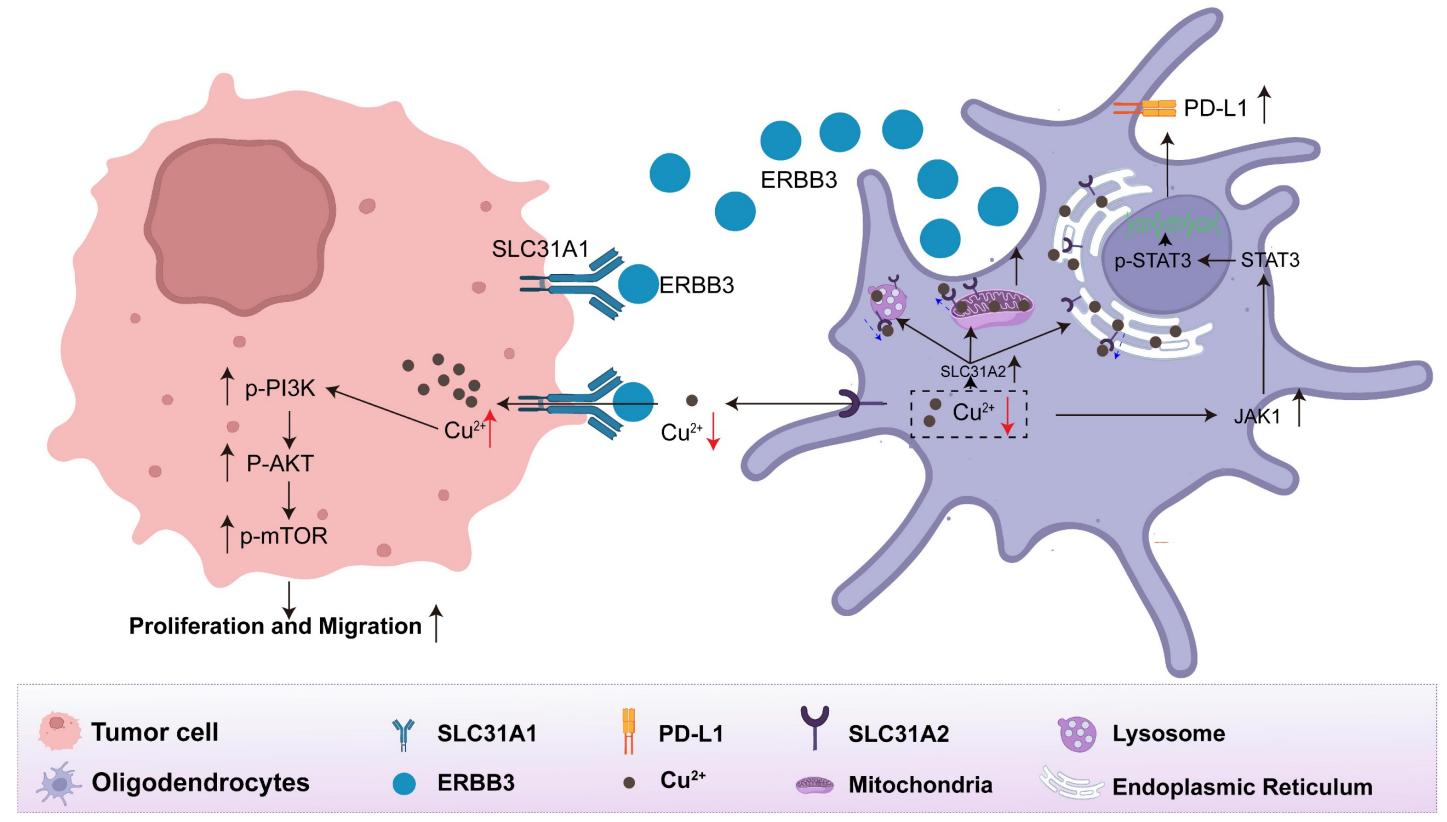
** The interaction between oligodendrocytes and tumor cells via the secretory ERBB3 and SLC31A1 axis**.

Collectively, we discovered that oligodendrocytes facilitate competitive absorption of copper from the tumor microenvironment into tumor cells via the secretory ERBB3 binding to SLC31A1 on the surface of tumor cells. This, in turn, stimulates the downstream PI3K-Akt-mTOR pathway in tumor cells and activates the JAK-STAT pathway within oligodendrocytes to remodel the immunosuppressive microenvironment. To visually convey these insights, we present a schematic diagram that elucidates the functional role and regulatory mechanisms of oligodendrocyte-secreted ERBB3 within the lung cancer brain metastatic microenvironment, as depicted in **Figure 7**.

## Discussion

LCBM typically progresses rapidly, shows an increasing incidence rate, and generally responds poorly to treatment 44. The unique characteristics of the brain microenvironment, along with the restrictive nature of the blood-brain barrier, render most drugs ineffective once metastasis is established 45. Drawing on the foundational seed-soil theory, it is recognized that the dynamic interplay within the brain tumor microenvironment plays a critical role in the progression of brain metastases and drug resistance 46. Thus, therapies specifically designed to target this microenvironment are crucial in the treatment of brain metastases. Previous studies have demonstrated that during the development and progression of brain metastases, tumor cells exhibit a diverse range of reciprocal interactions with various cell types, including vascular endothelial cells 7, neurons 6, astrocytes 10, microglia 47, macrophages 48 and neutrophils 49, 50. These interactions supported the colonization and growth of tumor cells and facilitate their immune evasion 46, 51.

Previous investigations employing scRNA-seq have provided clues that oligodendrocytes are present in LCBM 21-24. Our analysis revealed that oligodendrocytes were detected in at least one-third of LCBM, predominantly localized at the margins of metastatic lesions. While our study primarily focuses on the role of oligodendrocytes, we acknowledge that other CNS resident cells, such as astrocytes and microglia, are also key components of the metastatic niche and likely contribute to tumor progression through various mechanisms. It is critical to emphasize that cell proportions derived from scRNA-seq data may not accurately reflect tissue-level distributions, as these values are subject to variability due to factors such as lesion size at resection, tumor boundary clarity, and surgical completeness. In normal brain tissue, oligodendrocytes constitute 45%-75% of glial cells, with particularly high abundance in white matter, where they play a pivotal role in maintaining axonal myelin integrity 19. However, brain metastases from lung cancer predominantly localize at the gray-white matter junction, suggesting a potential abundance of oligodendrocytes in the peri-metastatic regions 20. The traditional view of oligodendrocytes as terminally differentiated, long-lived cells responsible for axonal myelination implies limited migratory capacity 52. Whether the oligodendrocytes observed at the tumor periphery arise from in situ differentiation of oligodendrocyte precursor cells (OPCs) or are recruited in a mature state through tumor-derived signaling remains unresolved. Addressing this question will require further mechanistic investigations into the interplay between neurodevelopmental processes and the tumor microenvironment.

In this study, we found it particularly intriguing that oligodendrocytes abundantly express ERBB3, with a notable increase in ERBB3 secretion when co-cultured with brain metastatic tumor cells. This observation was especially relevant in the context of LCBM, where ERBB3 secretion in the CSF of patients with LCBM was significantly higher compared to patients with gliomas or benign tumors. Furthermore, high ERBB3 expression is associated with poorer overall survival in our cohort, an association particularly pronounced in the EGFR-mutant subgroup, suggesting its potential as a prognostic biomarker. However, given the retrospective nature and remaining heterogeneity of the cohort, especially across molecular subtypes, this finding requires validation in larger, prospective, and uniformly treated patient sets.

Previous studies have demonstrated that ERBB3 is frequently overexpressed in LCBM across multiple cancer types and contributes to tumor resistance 27, 34, although the underlying mechanisms have remained unclear. Our research provided novel insights at the single-cell level, revealing that oligodendrocytes secreted substantial amounts of ERBB3, which could account for its high expression in brain metastasis of various cancers. Moreover, the interaction between secreted ERBB3 and SLC31A1 on the surface of tumor cells appeared to regulate copper ion homeostasis, potentially mediating ERBB3's role in promoting tumor resistance.

Metastatic tumors generally compete with surrounding microenvironmental cells for various nutrients to satisfy their rapid proliferation and survival needs, a phenomenon especially pronounced in nutrient-deficient microenvironments of brain metastasis 53-55. Chi *et al.* reported that disseminated cancer cells in brain fluids sequestered iron from macrophages to support metastatic survival 8. Indeed, several studies indicated that tumor tissues exhibit a greater demand for copper ions compared to healthy tissues 42. As an essential element within the human body, copper ions are vital for rapid cellular division, thus cancer cells require more copper than non-dividing cells. Elevated copper concentrations have been observed in animal models and in the tumors or serum of patients with various cancers, including breast cancer 56, lung cancer 57 and gastrointestinal cancer 58, 59.

In the brain—the second most copper-rich organ of the human body—copper serves as a crucial trace metal element 60. It serves as an essential cofactor in enzymatic processes, facilitating or participating in electron transfer reactions that are fundamental to various biochemical pathways, including mitochondrial energy production, tyrosine and neurotransmitter metabolism, redox homeostasis, and extracellular matrix remodeling 39. Our analysis of single-cell sequencing data from LCBM demonstrated that, in comparison to primary lung cancer cells, brain metastatic tumor cells showed significant enrichment in gene sets associated with copper ion metabolism, oxidative phosphorylation and cell cycle regulation. This implies that the accumulation of copper ions in metastatic tumor cells within the brain microenvironment does not initiate cell death. Instead, it seems to foster tumor growth and progression by supplying essential nutrients. Naturally, these findings necessitate further experimental validation.

Importantly, the clinical significance of ERBB3 has been substantiated through Phase I/III clinical trials (NCT03255070) evaluating the efficacy of ERBB3-directed ADC, in patients with refractory EGFR-mutated NSCLC (U31402-A-U102/NCT03255070/NCT05338970) 29-31. However, the substantial expression of ERBB3 in oligodendrocytes may potentially mediate off-target effects of HER3-targeted ADC therapies, warranting comprehensive preclinical investigations to elucidate its safety profile.

In summary, our comprehensive molecular analysis of the interplay between brain metastatic cells and oligodendrocytes in the LCBM microenvironment uncovered a novel ERBB3-SLC31A1-'copper homeostasis'-PI3K-Akt-mTOR signaling axis. While our data demonstrate that ERBB3 promotes metastatic progression through the activation of PI3K-Akt-mTOR signaling, we cannot exclude the possibility that additional, complementary molecular mechanisms may also contribute to its pro-tumorigenic effects. Significantly, our research highlighted the active role of oligodendrocytes in modulating tumor behavior through ERBB3 secretion, which further influenced copper ion distribution within the microenvironment and promoted tumor progression. Consequently, targeting ERBB3 and copper homeostasis presented promising therapeutic opportunities for patients with LCBM.

## Supplementary Material

Supplementary figures.

## Figures and Tables

**Table 1:** Baseline Characteristics of Lung Cancer Patients with Brain Metastasis

Characteristics	Whole cohort	High ERBB3	Low ERBB3	*P* value
n	80	40	40	
Gender, n (%)				0.648
Male	32.0 (40.0%)	25 (31.2%)	23 (28.7%)	
Female	48.0 (60.0%)	15 (18.8%)	17 (21.2%)	
Age, mean ± sd	61.5 (52.5, 66.0)	62.7 ± 7.4082	55.85 ± 10.553	0.001
EGFR, n (%)				0.370
0	38.0 (47.5%)	21 (26.2%)	17 (21.2%)	
1	42.0 (52.5%)	19 (23.8%)	23 (28.7%)	
ALK, n (%)				0.499
0	70.0 (87.5%)	36 (45%)	34 (42.5%)	
1	10.0 (12.5%)	4 (5%)	6 (7.5%)	
location, n (%)				0.292
Supratentorial	69.0 (86.3%)	33 (41.2%)	36 (45%)	
Subatentorial	3.0 (3.8%)	1 (1.2%)	2 (2.5%)	
Supratentorial and subatentorial	8.0 (10.0%)	6 (7.5%)	2 (2.5%)	
Number of brain metastasis, n (%)				0.052
1	54.0 (67.5%)	22 (27.5%)	32 (40%)	
2	18.0 (22.5%)	13 (16.2%)	5 (6.2%)	
3	8.0 (10.0%)	5 (6.2%)	3 (3.8%)	
Primary tumor resection surgery, n (%)				0.217
0	57.0 (71.3%)	26 (32.5%)	31 (38.8%)	
1	23.0 (28.8%)	14 (17.5%)	9 (11.2%)	
Postoperation radiotherapy, n (%)				0.501
1	37 (46.3%)	20 (25%)	23 (28.7%)	
0	43 (53.8%)	20 (25%)	17 (21.2%)	
Postoperative targeted therapy, n (%)				0.237
0	27 (33.8%)	16 (20%)	11 (13.8%)	
1	53 (66.3%)	24 (30%)	29 (36.2%)	
CYFRA21, median (IQR)	2.9 (2.1, 5.7)	2.895 (2.2875, 4.5975)	3.035 (1.93, 5.7875)	0.881
CEA, median (IQR)	6.5 (2.7, 19.7)	5.91 (2.95, 34.26)	6.875 (2.375, 12.12)	0.465
AFP, median (IQR)	2.8 (1.8, 3.7)	3.015 (2.2275, 3.9025)	2.45 (1.6875, 3.315)	0.260
CA15, median (IQR)	15.8 (8.9, 23.5)	15.795 (9.18, 20.075)	15.8 (8.835, 26.935)	0.458
CA19-9, median (IQR)	14.8 (8.1, 26.9)	14.465 (8.315, 30.827)	14.93 (7.7775, 26.36)	0.881
CA125, median (IQR)	20.8 (13.0, 49.9)	19.2 (13.5, 50.035)	26.5 (12.332, 49.855)	0.965
OS, median (IQR)	473.0 (242.0, 697.0)	350 (125.25, 610)	572.5 (397, 756.25)	< 0.001

**Table 2:** Univariate and multivariate cox regression analyses of overall survival in the Brain metastasis cohor (n = 80).

Characteristics	Total (N)	Univariate analysis			Multivariate analysis	
		Hazard ratio (95% CI)	P value		Hazard ratio (95% CI)	P value
Gender	80					
Male	48	Reference			Reference	
Female	32	0.469 (0.229 - 0.960)	0.038		0.886 (0.370 - 2.123)	0.786
Age	80	1.050 (1.009 - 1.093)	0.016		1.037 (0.983 - 1.094)	0.183
EGFR	80					
0	38	Reference				
1	42	0.616 (0.319 - 1.192)	0.150			
ALK	80					
0	70	Reference				
1	10	1.029 (0.396 - 2.673)	0.953			
location	80					
Supratentorial	69	Reference				
Subatentorial	3	1.344 (0.319 - 5.668)	0.687			
Supratentorial and subatentorial	8	1.791 (0.624 - 5.138)	0.279			
Number of brain metastasis	80					
1	54	Reference			Reference	
2	18	1.418 (0.625 - 3.214)	0.403		1.943 (0.786 - 4.805)	0.150
3	8	3.774 (1.587 - 8.974)	0.003		1.370 (0.538 - 3.490)	0.509
Primary tumor resection surgery	80					
0	57	Reference				
1	23	0.538 (0.223 - 1.297)	0.167			
Post-operation radiotherapy	80					
1	43	Reference			Reference	
0	37	2.700 (1.362 - 5.354)	0.004		1.697 (0.782 - 3.681)	0.181
Postoperative targeted therapy	80					
0	27	Reference			Reference	
1	53	0.148 (0.074 - 0.295)	< 0.001		0.159 (0.066 - 0.386)	< 0.001
ERBB3	80					
High	40	Reference			Reference	
Low	40	0.262 (0.127 - 0.542)	< 0.001		0.337 (0.148 - 0.771)	0.010

## Data Availability

The data sets used in this investigation are available through public repositories.
