# Supplementary material for: Oligodendrocyte-secreted ERBB3 Mediates the Competitive Uptake of Copper Ions by Tumor Cells to Promote Brain Metastasis in Lung Cancer
Source: Int J Biol Sci. 2026 Mar 25;22(7):3580–99. doi: 10.7150/ijbs.127108 (PMC13086005; doi:10.7150/ijbs.127108)
Supplement: Supplementary file 1 — Supplementary figures. [file ijbsv22p3580s1.pdf]

Supplementary Materials for

Oligodendrocyte-secreted ERBB3 Mediates the Competitive Uptake of Copper Ions by Tumor Cells to Promote Brain Metastasis in Lung Cancer

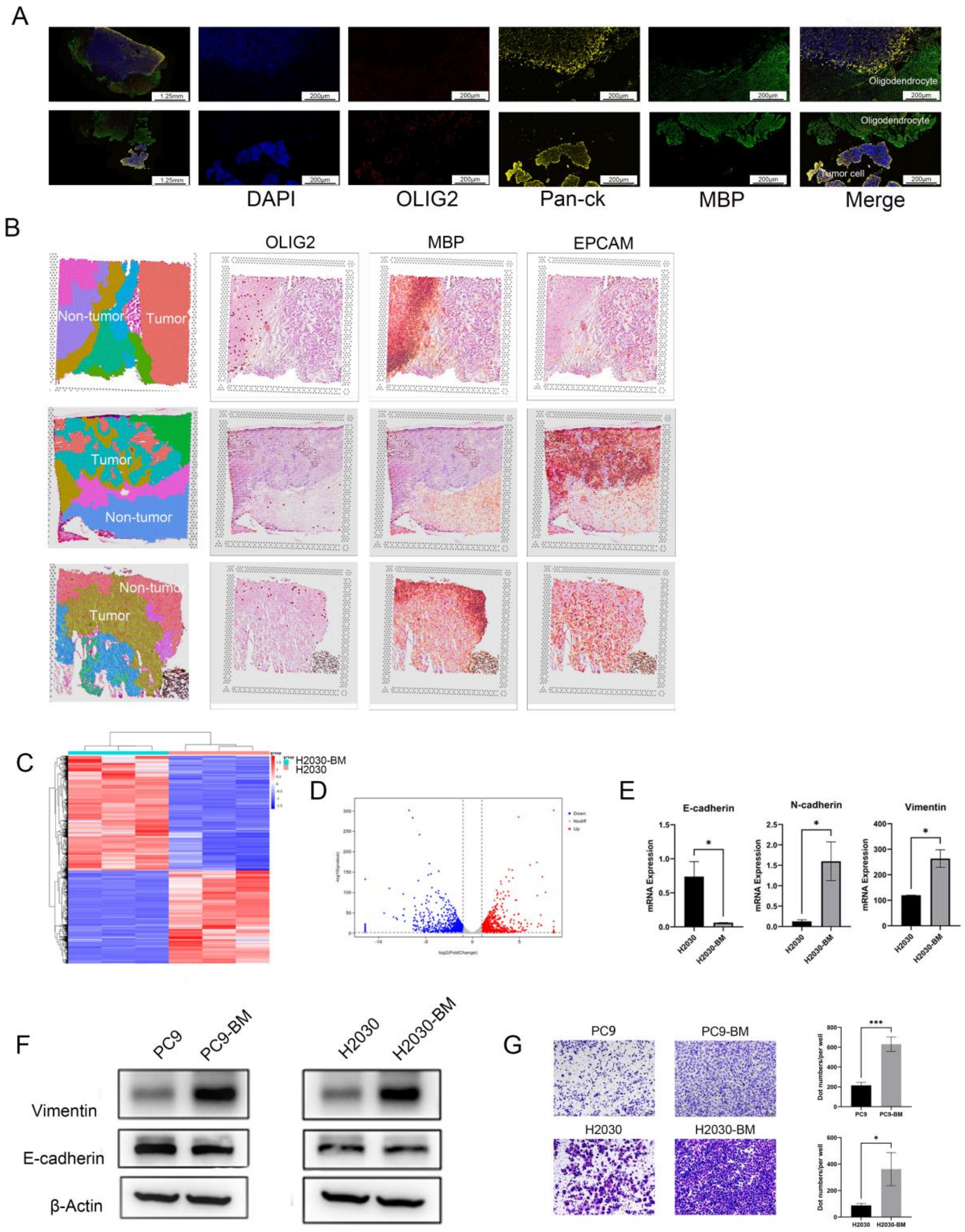

Figure S1 (A) Immunofluorescence staining analysis of the spatial distribution of oligodendrocytes in lung cancer brain metastases. (B) Spatial transcriptomic analysis of lung cancer brain metastasis specimens reveals the spatial distribution of oligodendrocytes. (C-E) Volcano map of differential gene and expression difference of EMT pathway-related molecules between high brain metastatic cells and

parental cells. (F) Protein expression analysis for EMT pathway-related analysis was performed using western blot. (G) The difference in invasive ability between high brain metastatic cells and parental cells was compared using transwell assay.

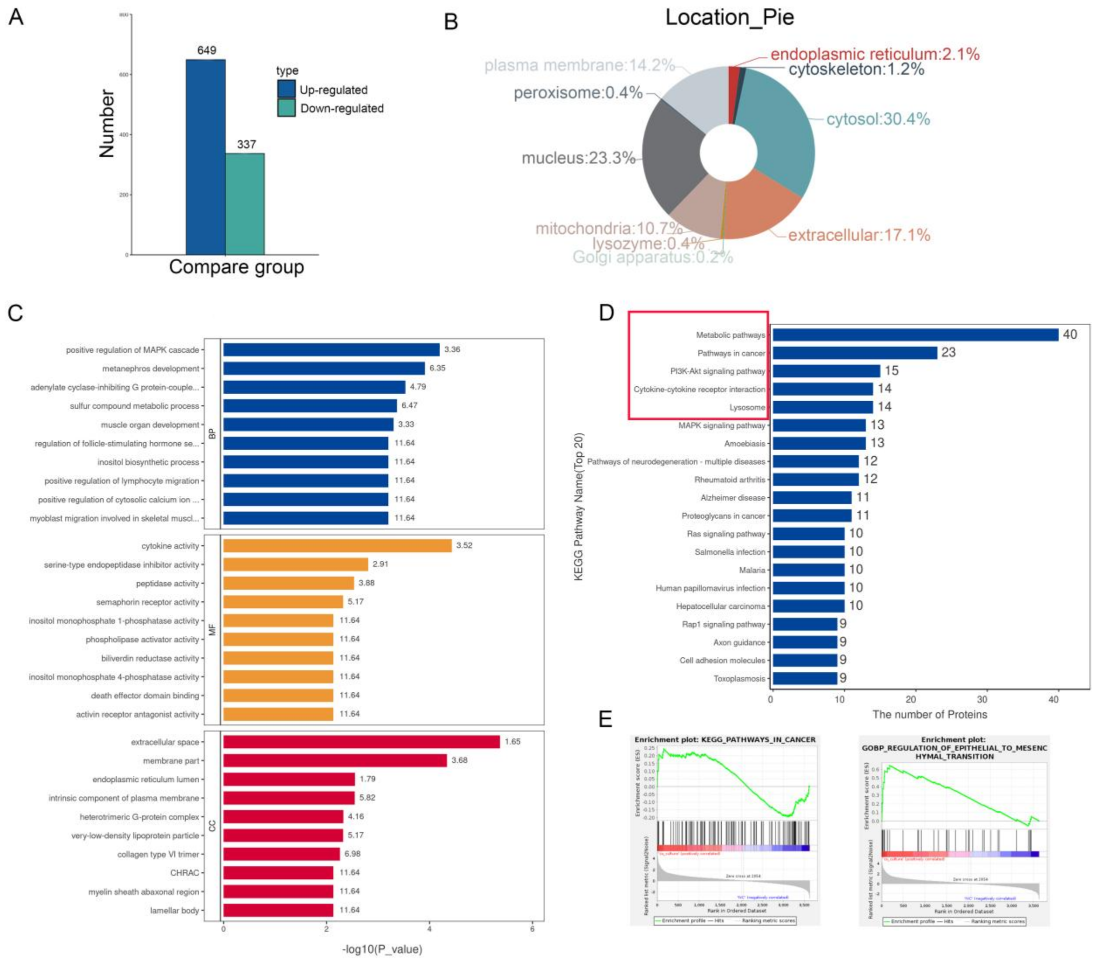

Figure S2 Pathway enrichment analysis of secretory proteomics in oligodendrocytes.(A) Histogram depicting the number of differentially expressed proteins. (B) Pie chart illustrating the cellular distribution of differentially expressed proteins. (C) GO enrichment analysis of differentially secreted proteins of oligodendrocytes after co-culture with tumor cells. (D) KEGG enrichment analysis of differentially secreted proteins of oligodendrocytes after co-culture with tumor cells. (E) GSEA enrichment analysis of differentially secreted proteins of oligodendrocytes after co-culture with tumor cells.

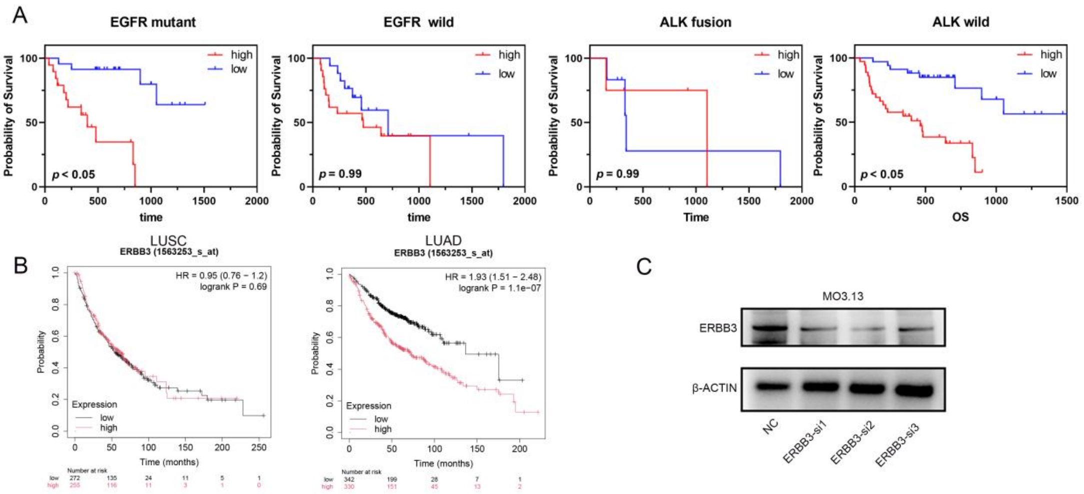

Figure S3 (A) Subgroup analysis to evaluate the prognostic value of ERBB3. (B) Analysis of the prognostic value of ERBB3 in the public database. (C) siRNA was used to knock down ERBB3 in MO3.13. Western blot was employed to detect the specificity of the ERBB3 antibody.

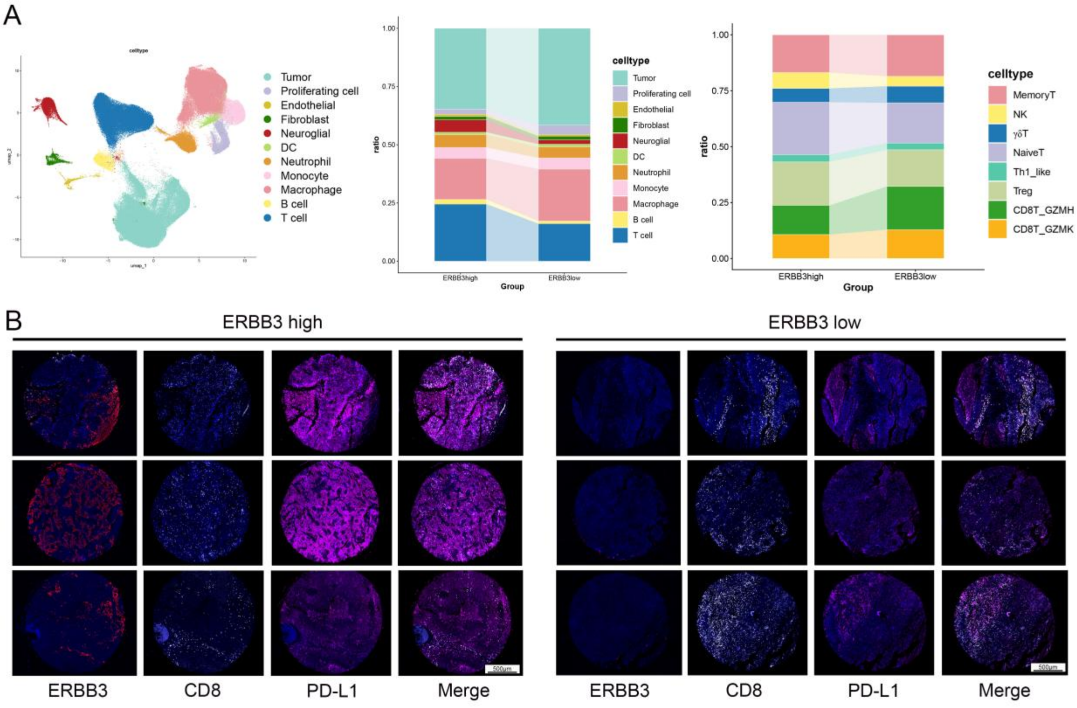

Figure S4 (A) Based on the high or low expression levels of ERBB3 in oligodendrocytes, cell clustering analysis was conducted at the single-cell level. (B) Validation of the relationship between the immune microenvironment and the expression of ERBB3 in oligodendrocytes in lung cancer brain metastasis samples.

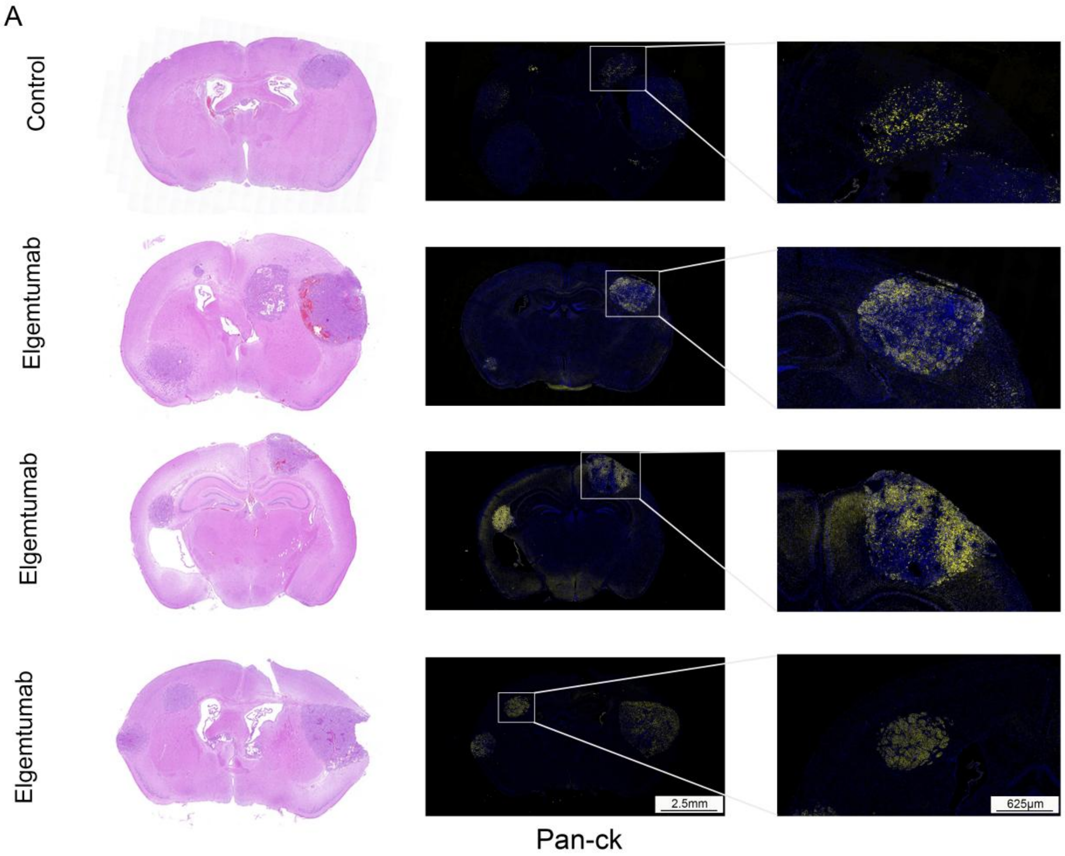

Figure S5 (A) Immunofluorescence staining was used to verify the intracranial metastasis in the animal model.
